# Middle Cerebral Artery Bifurcation Geometry and Aneurysm Formation: A Propensity Score-Matched 3D Rotational Morphometry Study

**DOI:** 10.3390/diagnostics16152312

**Published:** 2026-07-23

**Authors:** Shin Hyuck Bang, Min Kyun Na, Hyoung-Joon Chun, Hyeong-Joong Yi, Young-Jun Lee, Sang Hyung Lee, Jaiyoung Ryu, Simon Song, Kunhee Han, Kyu-Sun Choi

**Affiliations:** 1 Department of Neurosurgery, Hanyang University Medical Center, Hanyang University College of Medicine, Seoul 04763, Republic of Korea; bang0523bang@gmail.com (S.H.B.); tdy815@hanyang.ac.kr (H.-J.C.);; 2 Center for Precision Medicine Platform Based-on Smart Hemo-Dynamic Index, Seoul 04763, Republic of Korea; nslee@snu.ac.kr (S.H.L.); jryu@korea.ac.kr (J.R.);; 3 Department of Radiology, Hanyang University Medical Center, Hanyang University College of Medicine, Seoul 04763, Republic of Korea; 4 Department of Neurosurgery, Jeju National University Hospital, Jeju National University College of Medicine, Jeju 63241, Republic of Korea; 5 Department of Mechanical Engineering, Korea University, Seoul 02841, Republic of Korea; 6 Department of Mechanical Convergence Engineering, Hanyang University, Seoul 04763, Republic of Korea

**Keywords:** angiography, digital subtraction, intracranial aneurysm, middle cerebral artery

## Abstract

**Background/Objectives:** The middle cerebral artery (MCA) bifurcation is a common site of intracranial aneurysms. We evaluated associations between MCA bifurcation geometry and aneurysm presence and explored relationships between rupture and aneurysm size using a standardized 3D morphometry pipeline. **Methods:** This single-center, retrospective, propensity score-matched case–control study included 133 MCA bifurcation aneurysms and 266 matched controls, with bifurcation and sac-level geometry quantified using 3D rotational DSA. Associations with aneurysm presence were evaluated using generalized estimating equation (GEE) logistic regression clustered by patient ID. The discriminatory ability of individual geometric markers was assessed using ROC-AUC. **Results:** After matching, baseline covariates were balanced. In univariate comparisons, aneurysm-bearing bifurcations had a wider angle between the two M2 branches (opening angle), smaller dominant and non-dominant M1–M2 branch angles, smaller parent diameters and cross-sectional areas, a higher opening angle-to-parent diameter ratio (opening-to-caliber index), and lower parent-to-dominant-branch and daughter-branch diameter ratios. In multivariable GEE analysis, a larger opening angle, smaller dominant-branch angle, and lower D1/D2 ratio were independently associated with aneurysm presence; each 5° increase in opening angle corresponded to approximately 40% higher odds. The opening-to-caliber index showed the numerically highest single-marker ROC-AUC but did not remain independently associated with aneurysm presence after adjustment. Among the aneurysms, rupture was not associated with bifurcation geometry; ruptured aneurysms were larger. Across size strata, the opening angle and opening-to-caliber index increased, and the dominant-branch angle decreased. **Conclusions:** MCA bifurcation aneurysm presence was associated with a wider, more asymmetric bifurcation geometry, whereas rupture appeared more closely related to sac-level morphology.

## 1. Introduction

Intracranial aneurysms are common cerebrovascular lesions and a major cause of subarachnoid hemorrhage (SAH), which carries substantial morbidity and mortality [1,2,3,4,5]. The middle cerebral artery (MCA) bifurcation is among the most frequent sites of aneurysm formation, accounting for approximately one-fifth of all cerebral aneurysms [6,7]. Aneurysms preferentially arise at bifurcations and curvatures, where pulsatile flow division generates elevated wall shear stress (WSS) and spatial WSS gradients (WSSGs) at the apical wall; the MCA bifurcation is a representative site of this hemodynamic loading pattern [5,6,7,8,9,10].

Prior work suggests that aneurysm formation is associated with wide and asymmetric bifurcations characterized by imbalanced parent-to-branch caliber ratios and branching angles [6,7,8,9]. These geometric configurations may direct inflow jets toward the bifurcation apex and increase wall shear stress and wall shear stress gradients, potentially predisposing the apical wall to aneurysm initiation [5,8,11,12]. In contrast, rupture risk has been more consistently linked to sac-level characteristics, including larger size and irregular surface morphology [1,3,4,13,14,15]. Whether the same bifurcation geometries that predispose to aneurysm formation are also related to rupture remains uncertain [1,3,4,13,14,15].

Most studies have examined either formation-related bifurcation geometry by comparing aneurysmal versus non-aneurysmal MCAs [6,7,9] or rupture-related sac morphology within aneurysm cohorts. Few studies have evaluated the formation and rupture together at the MCA bifurcation, and size-stratified patterns of bifurcation geometry remain incompletely characterized.

We analyzed MCA bifurcation aneurysms and propensity-matched controls using standardized three-dimensional (3D) rotational digital subtraction angiography morphometry. The primary objective was to identify MCA bifurcation geometries independently associated with the presence of aneurysms. Secondary case-only analyses evaluated the association between rupture status and aneurysm size strata.

## 2. Materials and Methods

### 2.1. Study Design and Population

This single-center, retrospective, propensity-score-matched, case–control study was conducted at a tertiary academic center from January 2017 to December 2022 using an Azurion 7 B20 angiography system (Philips Medical Systems Nederland B.V., Best, the Netherlands). The study protocol was approved by the institutional review board (No. 2023-04-028) and adhered to the Declaration of Helsinki. The requirement for informed consent was waived because of the retrospective design of the study.

We retrospectively identified adults who underwent 3D rotational DSA for cerebrovascular treatment during the study period. We included patients with at least one MCA bifurcation aneurysm with image quality suitable for quantitative morphometry (*n* = 133), defined as a saccular lesion arising from the M1–M2 bifurcation apex and incorporating the origin of one or both M2 branches; aneurysms arising purely from M2 trunks or more distal segments were excluded, following previous studies [6,7]. Controls were selected from the same pool of patients and had no MCA aneurysms on angiographic review. Cases and controls were propensity-score-matched 1:2 on age, sex, body mass index (BMI), and hypertension [1,3,4]. Smoking was not included among the matching variables because the matching strategy was pre-specified to balance the major demographic and vascular comorbidity factors. The potential impact of residual confounding factors related to smoking was addressed through sensitivity analyses and is acknowledged as a limitation.

The sample size was determined by the number of eligible cases available during the study period, and no sample size calculation was performed in advance because of the retrospective design.

### 2.2. Clinical Variables and Clustering

Since the analyses were performed at the aneurysm level, patients with multiple MCA bifurcation aneurysms could have contributed more than one aneurysm to the case–cohort. The baseline clinical characteristics included routine vascular risk factors and medication use, as in previous studies [1,3,4]. All regression analyses accounted for patient-level clustering, and rupture status was not used for matching.

### 2.3. Imaging and Morphometry

**Figure 1:**
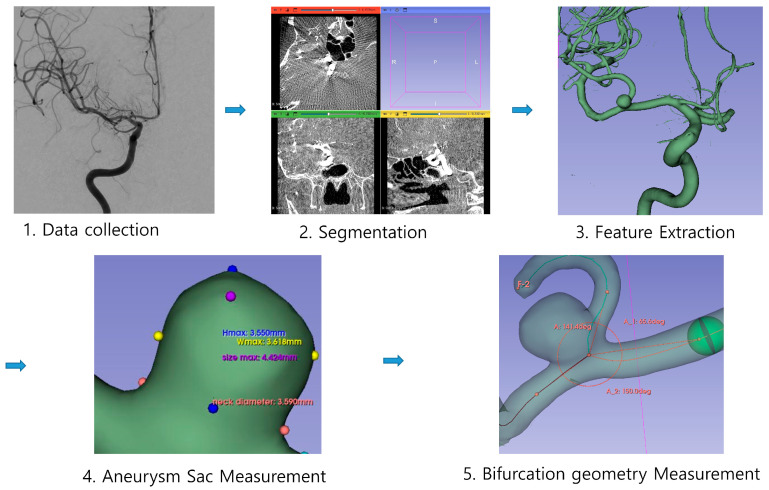
Radiomics-style measurement workflow based on three-dimensional rotational digital subtraction angiography. Blue arrows indicate the workflow from data collection and segmentation to feature extraction, followed by parallel aneurysm sac and bifurcation geometry measurements. Colored points and lines indicate anatomical landmarks and measurement axes used to quantify aneurysm size, neck diameter, bifurcation angle, M1–M2 angles, and vessel diameters.

Figure 1 illustrates the workflow of 3D rotational DSA acquisition through segmentation, feature extraction, and bifurcation- and sac-level measurements. Source DICOM data were anonymized and reconstructed into 1 × 1 × 1 mm^3^ isotropic volumes. Aneurysm and vessel segmentation and shape-based measurements were performed using 3D Slicer (version 5.10.0; Slicer Community; https://www.slicer.org). Vessel centerlines were extracted using the SlicerVMTK extension (Vascular Modeling Toolkit; https://www.vmtk.org), and M1 and M2 trajectories were used to define the bifurcation angles and diameters [16,17]. The surface area and convex-hull volume were obtained using the Segment Statistics module.

**Figure 2:**
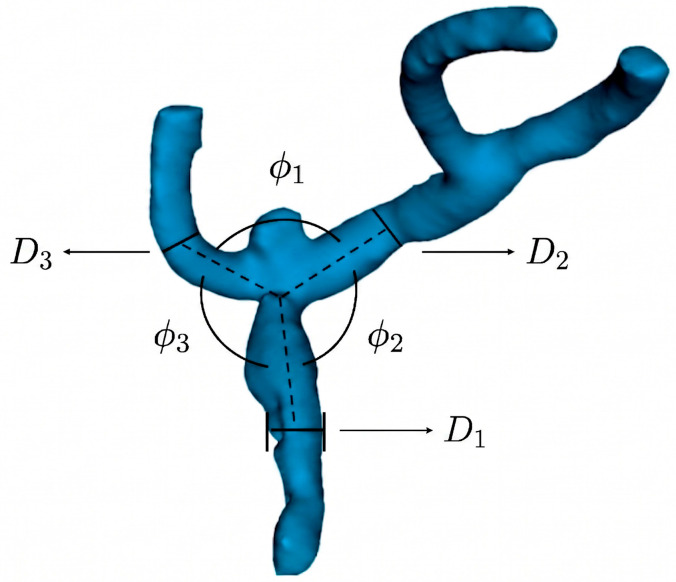
Schematic illustration of MCA bifurcation geometry, including the bifurcation angle (ϕ1), M1–dominant M2 angle (ϕ2), M1–non-dominant M2 angle (ϕ3), M1 diameter (D1), dominant M2 diameter (D2), and non-dominant M2 diameter (D3).

At the bifurcation level, we measured the opening angle (ϕ1), dominant and non-dominant M1–M2 branch angles (ϕ2, ϕ3), and parent and daughter diameters (D1–D3). The dominant M2 branch was the daughter branch with the largest diameter. The derived variables included D1/D2, D1/D3, D3/D2, ϕ1/D1, ϕ3/ϕ2, and the parent cross-sectional area (Supplementary Table S1). Figure 2 shows the definitions of angle and diameter. At the sac level, we quantified the maximum aneurysm size, neck diameter, height, width, volume, convex-hull volume, sac angles, and surface irregularity/bleb using prior definitions [13,14] (Supplementary Table S2). Two experienced neurosurgeons independently performed all measurements after calibration using a pre-specified protocol and centerline-guided axes, blinded to clinical variables and study hypotheses. Inter-rater agreement was excellent, with intraclass correlation coefficients > 0.85 for all primary parameters. Reproducibility was assessed across all bifurcation-level measurements in the matched cohort (n = 399 bifurcations) and all sac-level measurements in the aneurysm cohort (n = 133 aneurysms), as applicable. Intraclass correlation coefficients (two-way random-effects, absolute-agreement model) were calculated from the two raters’ independent measurements prior to consensus. When discrepancies were identified, the two neurosurgeons jointly re-reviewed the centerline landmarks, branch assignment, and measurement planes to reach consensus, and the consensus values were used in the final analyses.

### 2.4. Propensity-Score Matching

Propensity scores for having an MCA bifurcation aneurysm were estimated using logistic regression. One-to-two nearest-neighbor matching without replacement was applied using the MatchIt package in R (version 4.5.2; R Foundation for Statistical Computing, Vienna, Austria). Covariate balance was assessed using standardized mean differences (SMDs), with |SMD| < 0.10 considered acceptable. Detailed pre- and post-matching SMDs are presented in Supplementary Table S3. All covariates showed good balance after matching.

### 2.5. Statistical Analysis

All analyses were conducted in R version 4.5.2 (R Foundation for Statistical Computing, Vienna, Austria). Continuous variables were summarized as mean ± standard deviation or median [interquartile range, IQR] and compared using Student’s *t*-test or the Wilcoxon rank-sum test, as appropriate. Categorical variables are summarized as n (%) and compared using the χ^2^ test or Fisher’s exact test. All tests were two-sided, and statistical significance was set at α = 0.05. Where applicable, effect sizes with 95% confidence intervals (CIs) were reported. All regression analyses accounted for within-patient dependence by clustering on patient ID. There were no missing values for any of the variables included in the reported analyses; therefore, no imputation was performed. Receiver operating characteristic (ROC) analyses were summarized using the area under the curve (AUC) with 95% CIs. Pre-specified sensitivity analyses were performed to assess the robustness of the formation-related findings (Supplementary Table S8). Baseline characteristics after propensity-score matching and univariable comparisons of bifurcation-level geometry are summarized in Table 1 and Table 2, respectively.

### 2.6. Analysis of Aneurysm Presence

**Figure 3:**
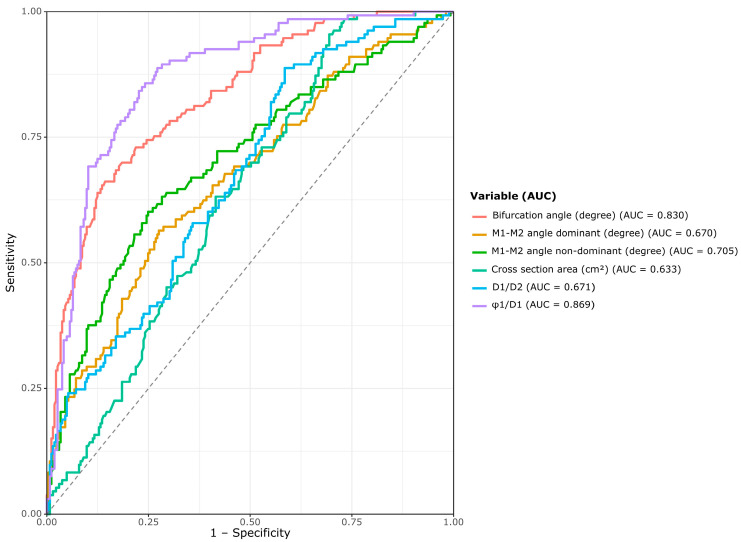
ROC curves of single geometric markers for MCA bifurcation aneurysm presence. The dashed diagonal line represents the no-discrimination reference line (AUC = 0.50). AUCs with 95% confidence intervals are shown in Table 4.

Generalized estimating equation (GEE) logistic regression models with an independence working correlation structure were fitted in the matched cohort, with clustering on patient ID to account for within-patient dependence. The pre-specified geometric predictors were ϕ1, ϕ2, ϕ3, D1/D2, ϕ1/D1, and parent cross-sectional area, selected from prior morphometric and hemodynamic research [5,6,7,10,18,19,20,21]. Models were fitted on the original measurement scale; Table 3 reports aORs per 1° for angles and per 1.0-unit for D1/D2 and ϕ1/D1, with selected text estimates rescaled for interpretability. Model parsimony was assessed using stepwise Akaike information criterion selection. Discrimination was evaluated using ROC-AUCs (Figure 3 and Table 4).

### 2.7. Size Stratification and Rupture Analyses

Rupture-status and size-stratified analyses were performed within the aneurysm cohort, and an additional small-aneurysm subgroup analysis was performed against matched controls. First, ruptured and unruptured aneurysms were compared with respect to baseline clinical characteristics, sac-level morphology, and bifurcation-level geometry (Supplementary Table S5 and Table 5 and Table 6).

Second, aneurysms were stratified into three size groups: small <4 mm, medium 4–6.9 mm, and large ≥7 mm. These thresholds were based on prior natural-history data and commonly used clinical criteria. In prospective cohorts of small unruptured aneurysms (<5 mm), aneurysm size ≥ 4 mm has been reported to be associated with a higher rupture risk [22]. Size ≥ 7 mm is also a widely used threshold at which rupture risk increases in large Japanese and pooled Western cohorts [1,2,3,4].

Size groups were compared with respect to baseline clinical characteristics, bifurcation-level geometry, and sac-level morphology using ANOVA or Kruskal–Wallis tests for continuous variables and χ^2^ or Fisher’s exact tests for categorical variables, with Bonferroni-adjusted pairwise comparisons where appropriate (Supplementary Table S6 and Table 7 and Table 8). A subgroup analysis comparing small aneurysms (<4 mm) with controls was also performed using the same stepwise GEE approach to examine whether aneurysm-presence associations observed in the primary analysis were maintained in small lesions (Supplementary Table S7).

## 3. Results

Of the 782 patients treated for ruptured or unruptured intracranial aneurysms, 156 MCA aneurysms were identified. After excluding aneurysms arising from the M2 trunks or distal segments, incomplete 3D rotational DSA, and insufficient image quality, 133 MCA bifurcation aneurysms remained. From the same treated cohort, 266 controls without MCA aneurysms and with adequate M1–M2 visualization were selected by 1:2 propensity-score matching. The baseline covariates were well-balanced after matching (all standardized mean differences < 0.10; Table 1 and Supplementary Table S3). The operational definitions are provided in Supplementary Table S4.

**Table 1:** Baseline characteristics after 1:2 propensity-score matching.

Characteristics	Total (n = 399)	Control (n = 266)	Case (n = 133)	*p*
Age	60.15 ± 11.35	60.13 ± 12.47	60.20 ± 8.73	0.944
Sex (Male)	142 (35.6)	97 (36.5)	45 (33.8)	0.684
BMI	23.76 ± 3.58	23.69 ± 3.47	23.90 ± 3.79	0.584
HTN (%)	222 (55.6)	147 (55.3)	75 (56.4)	0.915
Diabetes mellitus (%)	32 (8.0)	22 (8.3)	10 (7.5)	0.948
Hyperlipidemia (%)	81 (20.3)	55 (20.7)	26 (19.5)	0.895
Heart disease (%)				0.800
Arrhythmia	7 (1.8)	6 (2.3)	1 (0.8)	
CAD	21 (5.3)	15 (5.6)	6 (4.5)	
HF	3 (0.8)	2 (0.8)	1 (0.8)	
Family Hx of CVD (%)	5 (1.3)	3 (1.1)	2 (1.5)	1.000
Family Hx of aneurysm (%)	10 (2.5)	7 (2.6)	3 (2.3)	1.000
Drinking (%)	141 (35.3)	89 (33.5)	52 (39.1)	0.317
Smoking (%)	102 (25.6)	63 (23.7)	39 (29.3)	0.273
Antiplatelet (%)	66 (16.5)	44 (16.5)	22 (16.5)	1.000
Anticoagulant (%)	1 (0.3)	1 (0.4)	0 (0.0)	1.000
Lipid_lowering agent (%)	83 (20.8)	57 (21.4)	26 (19.5)	0.760

HTN, hypertension; CAD, coronary artery disease; CVD, cerebrovascular disease.

### 3.1. Case–Control Differences in Geometry

Bifurcation-level geometry showed clear case–control contrasts (Table 2). Compared with controls, aneurysm-bearing bifurcations had larger opening angles ϕ1, smaller dominant and non-dominant M1–M2 branch angles ϕ2 and ϕ3, a smaller parent diameter D1 with a lower cross-sectional area, a smaller non-dominant-daughter diameter D3, a higher opening-to-caliber index ϕ1/D1, and lower D1/D2 and D3/D2 diameter ratios. In contrast, the angle ratio ϕ3/ϕ2 and the parent-to-non-dominant-branch ratio D1/D3 did not differ significantly.

**Table 2:** Bifurcation-level geometry in MCA bifurcation aneurysm vs. matched controls (univariable comparison).

Characteristics	Total (n = 399)	Control (n = 266)	Case (n = 133)	*p*
M1 diameter (mm)	2.51 ± 0.58	2.61 ± 0.63	2.31 ± 0.40	<0.001
Dominant M2 diameter (mm)	2.24 ± 0.46	2.23 ± 0.48	2.26 ± 0.41	0.604
Non-dominant M2 diameter (mm)	1.63 ± 0.41	1.66 ± 0.41	1.57 ± 0.38	0.026
Bifurcation angle (°)	115.01 ± 30.05	103.91 ± 27.43	137.21 ± 21.64	<0.001
M1–M2 angle dominant (°)	124.97 ± 22.74	129.90 ± 19.59	115.12 ± 25.35	<0.001
M1–M2 angle non-dominant (°)	102.77 ± 26.44	108.89 ± 24.47	90.53 ± 26.08	<0.001
Cross-sectional area (cm^2^)	0.06 ± 0.05	0.07 ± 0.06	0.05 ± 0.02	<0.001
φ3/φ2 (angle ratio)	0.87 ± 0.34	0.87 ± 0.30	0.86 ± 0.41	0.693
D3/D2 (diameter ratio)	0.74 ± 0.18	0.76 ± 0.18	0.71 ± 0.18	0.013
D1/D2	1.14 ± 0.26	1.19 ± 0.27	1.04 ± 0.20	<0.001
D1/D3	1.62 ± 0.52	1.65 ± 0.50	1.57 ± 0.55	0.215
φ1/D1	47.78 ± 16.67	41.01 ± 12.57	61.33 ± 15.62	<0.001

### 3.2. Independent Geometric Predictors

The final stepwise GEE model (Table 3) showed that a larger ϕ1 was associated with aneurysm presence (aOR 1.07 per 1°, 95% CI 1.04–1.10; *p* < 0.001), corresponding to approximately 40% higher odds per 5° increase. Larger ϕ2 was inversely associated with aneurysm presence (aOR 0.98 per 1°, 95% CI 0.97–1.00; *p* = 0.010), and lower D1/D2 was also associated with aneurysm presence (aOR 0.14 per 1.0-unit increase, 95% CI 0.03–0.72; *p* = 0.018). ϕ3 and ϕ1/D1 did not reach conventional statistical significance in the primary multivariable model (*p* = 0.052 and *p* = 0.064, respectively).

**Table 3:** GEE logistic regression for MCA bifurcation aneurysm presence.

Predictor (Unit)	Full Multivariable GEE aOR (95% CI)	*p*	Final Stepwise GEE aOR (95% CI)	*p*
Bifurcation angle, ϕ1 (per 1°)	1.07 (1.03–1.12)	0.001	1.07 (1.04–1.10)	<0.001
M1–dominant M2 angle, ϕ2 (per 1°)	0.98 (0.97–1.00)	0.009	0.98 (0.97–1.00)	0.010
M1–non-dominant M2 angle, ϕ3 (per 1°)	0.99 (0.97–1.00)	0.054	0.99 (0.97–1.00)	0.052
D1/D2 (per 1.0)	0.11 (0.00–18.70)	0.402	0.14 (0.03–0.72)	0.018
ϕ1/D1 (per 1.0)	0.96 (0.88–1.04)	0.315	0.95 (0.91–1.00)	0.064

### 3.3. Single Geometric Markers for Aneurysm Presence

In the single-marker ROC analyses, ϕ1/D1 showed the greatest discriminatory ability for aneurysm formation, followed by ϕ1; the remaining metrics showed moderate discrimination (Figure 3 and Table 4).

**Table 4:** ROC-AUCs of single geometric markers for MCA bifurcation aneurysm presence.

Variable	AUC (95% CI)
Bifurcation angle (°)	0.83 (0.789–0.872)
M1–M2 angle dominant (°)	0.67 (0.613–0.727)
M1–M2 angle non-dominant (°)	0.705 (0.649–0.761)
Cross-sectional area (cm^2^)	0.633 (0.578–0.687)
D1/D2	0.671 (0.617–0.725)
ϕ1/D1	0.869 (0.832–0.905)

### 3.4. Rupture-Stratified Comparisons

Within the aneurysm cohort, patients with ruptured aneurysms (n = 29) had a lower BMI (*p* = 0.002) and were more likely to be current smokers (*p* = 0.021) than those with unruptured aneurysms; other clinical covariates were similar between the groups (all *p* > 0.05; Supplementary Table S5). At the sac level, ruptured aneurysms were larger in maximum diameter, width, and height, whereas neck diameter, sac volume, convex-hull volume, convex-hull surface area, and sac angle were similar (Table 5). Surface irregularity and bleb formation were numerically more frequent in ruptured aneurysms, but these differences were not statistically significant (*p* = 0.09 and 0.21, respectively; Table 5). At the bifurcation level, we did not detect significant differences in geometric variables according to rupture status (all *p* > 0.20; Table 6).

**Table 5:** Case-only sac-level morphology by rupture status.

Characteristics	Unruptured (n = 104)	Ruptured (n = 29)	Case (n = 133)	*p*
Aneurysm max size (mm)	5.71 ± 3.21	7.53 ± 3.45	6.11 ± 3.34	0.015
Height maximum (mm)	4.10 ± 2.80	5.23 ± 2.64	4.35 ± 2.80	0.050
Width maximum (mm)	3.92 ± 2.55	5.16 ± 2.40	4.19 ± 2.56	0.019
Neck diameter (mm)	3.98 ± 1.77	4.09 ± 1.69	4.00 ± 1.74	0.764
Aneurysm volume (mm^3^)	125.47 ± 417.19	114.42 ± 139.33	123.06 ± 374.09	0.820
Convex hull volume (mm^3^)	129.51 ± 428.81	130.64 ± 158.46	129.76 ± 385.75	0.982
Convex hull surface area (mm^2^)	92.57 ± 152.70	118.94 ± 95.94	98.32 ± 142.36	0.261
Flow angle (°)	131.27 ± 22.58	136.42 ± 20.88	132.39 ± 22.24	0.254
Inclination angle (°)	84.94 ± 19.70	91.78 ± 18.39	86.43 ± 19.56	0.088
Vessel angle (°)	69.01 ± 27.83	76.62 ± 33.66	70.67 ± 29.23	0.271
Bleb formation (%)	25 (24.0)	11 (37.9)	36 (27.1)	0.210
Irregularity (%)	44 (42.3)	18 (62.1)	62 (46.6)	0.094

**Table 6:** Case-only bifurcation-level geometry by rupture status.

Variable	Unruptured (n = 104)	Ruptured (n = 29)	*p*
M1 diameter (mm)	2.33 ± 0.38	2.24 ± 0.45	0.352
Dominant M2 diameter (mm)	2.28 ± 0.41	2.16 ± 0.39	0.161
Non-dominant M2 diameter (mm)	1.58 ± 0.37	1.55 ± 0.44	0.750
Bifurcation angle (°)	136.70 ± 22.09	139.03 ± 20.18	0.593
M1–M2 angle dominant (°)	116.28 ± 25.55	110.97 ± 24.62	0.314
M1–M2 angle non-dominant (degree)	89.56 ± 25.36	94.02 ± 28.71	0.453
Cross-sectional area (cm^2^)	0.05 ± 0.02	0.05 ± 0.02	0.576
φ3/φ2 (angle ratio)	0.84 ± 0.40	0.92 ± 0.44	0.348
D3/D2 (diameter ratio)	0.71 ± 0.18	0.73 ± 0.20	0.615
D1/D2	1.04 ± 0.19	1.05 ± 0.23	0.767
D1/D3	1.57 ± 0.50	1.60 ± 0.71	0.813
φ1/D1	60.41 ± 15.36	64.62 ± 16.37	0.222

### 3.5. Size-Stratified Comparisons

**Figure 4:**
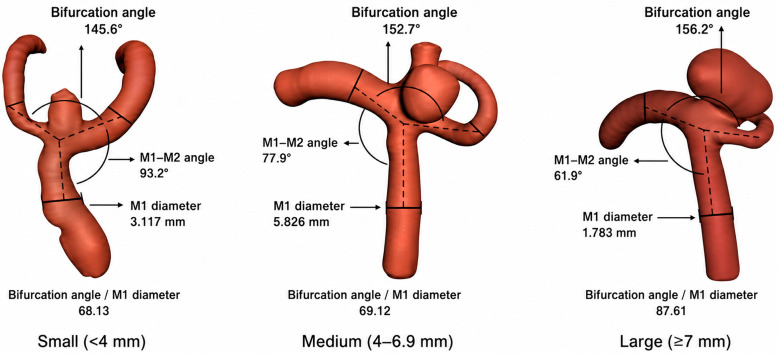
Size-stratified trends. Left-to-right: small (<4 mm), medium (4–6.9 mm), and large (≥7 mm).

In size-stratified analyses (small <4 mm, medium 4–6.9 mm, and large ≥7 mm), clinical characteristics did not differ across size groups (Supplementary Table S6), whereas bifurcation geometry and sac morphology varied across strata (Table 7 and Table 8 and Figure 4). At the bifurcation level, the opening angle ϕ1 increased with size (*p* = 0.033), the dominant-branch angle ϕ2 decreased (*p* < 0.001), and ϕ1/D1 increased (*p* = 0.026), whereas ϕ3, diameter ratios, and D1 cross-sectional area did not differ significantly (Table 7). As expected, sac-level size-related measures increased across size strata (all *p* < 0.001), and both bleb formation and overall surface irregularity also increased with size (both *p* < 0.001; Table 8).

**Table 7:** Case-only bifurcation-level geometry by aneurysm size.

Variable	Small (<4 mm) (n = 35)	Medium (4–6.9 mm) (n = 62)	Large (≥7 mm) (n = 36)	*p*
M1 diameter (mm)	2.39 ± 0.42	2.31 ± 0.35	2.24 ± 0.44	0.345
Dominant M2 diameter (mm)	2.23 ± 0.35	2.28 ± 0.43	2.25 ± 0.42	0.827
Non-dominant M2 diameter (mm)	1.54 ± 0.39	1.58 ± 0.35	1.58 ± 0.43	0.858
Bifurcation angle (°)	129.64 ± 18.29	139.70 ± 22.10	140.28 ± 22.63	0.033
M1-M2 angle dominant (°)	124.01 ± 19.91	118.30 ± 25.01	101.00 ± 25.44	<0.001
M1-M2 angle non-dominant (°)	93.47 ± 22.10	91.01 ± 25.78	86.85 ± 30.14	0.577
Cross-sectional area (cm^2^)	0.05 ± 0.02	0.05 ± 0.01	0.05 ± 0.02	0.761
φ3/φ2 (angle ratio)	0.79 ± 0.30	0.85 ± 0.45	0.93 ± 0.43	0.271
D3/D2 (diameter ratio)	0.70 ± 0.19	0.71 ± 0.16	0.72 ± 0.22	0.941
D1/D2	1.08 ± 0.20	1.03 ± 0.20	1.01 ± 0.18	0.236
D1/D3	1.65 ± 0.49	1.54 ± 0.49	1.57 ± 0.69	0.574
φ1/D1	55.92 ± 12.34	61.89 ± 13.33	65.62 ± 20.34	0.026

**Table 8:** Case-only sac-level morphology by aneurysm size.

Variable	Small (<4 mm) (n = 35)	Medium (4–6.9 mm) (n = 62)	Large (≥7 mm) (n = 36)	*p*
Aneurysm max size (mm)	3.08 ± 0.62	5.31 ± 0.79	10.42 ± 3.34	<0.001
Height maximum (mm)	2.17 ± 0.72	3.67 ± 0.99	7.63 ± 3.25	<0.001
Width maximum (mm)	2.32 ± 0.76	3.51 ± 0.90	7.16 ± 3.04	<0.001
Neck diameter (mm)	2.68 ± 0.52	3.74 ± 0.88	5.75 ± 2.21	<0.001
Aneurysm volume (mm^3^)	9.88 ± 6.10	39.20 ± 20.62	377.52 ± 659.66	<0.001
Convex hull volume (mm^3^)	10.55 ± 8.03	40.38 ± 21.11	399.57 ± 676.11	<0.001
Convex hull surface area (mm^2^)	23.75 ± 13.07	58.65 ± 19.61	239.13 ± 216.47	<0.001
Flow angle (°)	130.43 ± 20.69	131.83 ± 20.91	135.26 ± 25.99	0.685
Aneurysm inclination angle (°)	79.78 ± 16.68	86.17 ± 21.03	93.35 ± 17.57	0.006
Vessel angle (°)	73.68 ± 30.66	65.59 ± 27.97	76.47 ± 29.26	0.161
Bleb formation (%)	1 (2.9)	15 (24.2)	20 (55.6)	<0.001
Irregularity (%)	2 (5.7)	31 (50.0)	29 (80.6)	<0.001

In a pre-specified small-aneurysm subgroup analysis (<4 mm vs. controls), a larger bifurcation opening angle remained independently associated with aneurysm presence, whereas the other geometric variables were not retained in the final stepwise model (Supplementary Table S7).

## 4. Discussion

Our findings suggest that the presence of MCA bifurcation aneurysm is associated with bifurcation geometry, whereas rupture status appears to be more closely related to sac-level morphology than bifurcation geometry. In the matched analysis, aneurysm-bearing bifurcations were wider and more asymmetric than those in the controls. In analyses restricted to aneurysm cases, we did not detect significant associations between rupture status and bifurcation geometry, whereas sac size showed clearer separation between ruptured and unruptured aneurysms [1,2,4,13,14,15].

Unlike prior studies that examined either formation-related geometry in unmatched cohorts or rupture risk based on sac-level metrics alone [6,7,9], this study integrates a propensity-score-matched design with a comparative ranking of geometric discriminators and a size-stratified analysis, allowing us to distinguish geometric features that plausibly precede aneurysm formation from those that emerge secondary to aneurysm growth.

### 4.1. Formation-Associated Geometry at the MCA Bifurcation

Consistent with prior studies, aneurysms occurred at wide, asymmetric MCA bifurcations with imbalanced caliber and unequal branch angles [6,7,20,21]. In our matched cohort, aneurysm-bearing bifurcations showed wider bifurcation angles, more acute dominant-branch angles, lower parent-to-branch diameter ratios, and greater daughter-branch asymmetry than controls; ϕ1, ϕ2, and D1/D2 remained associated with aneurysm presence after multivariable adjustment. For interpretability, each 5° increase in opening angle corresponded to approximately 40% higher odds, whereas each 5° increase in the dominant-branch angle and each 0.1 increase in D1/D2 corresponded to approximately 10% and 18% lower odds, respectively. These findings are consistent with hemodynamic principles by which a wider opening angle and asymmetric branch calibers shift the impinging inflow jet away from the bifurcation centerline, concentrating WSS and WSSG at the apical wall—the region most susceptible to endothelial injury and aneurysm initiation in computational and in vivo hemodynamic studies [11,12,18,19,20,21]. Mechanistically, this interpretation assumes that MCA bifurcation geometry is a largely stable, pre-existing anatomical trait rather than a consequence of aneurysm development itself. This assumption is supported by prior observations that parent-vessel geometry tends to change only in relation to the aneurysm or its treatment (e.g., after stent-assisted coiling) rather than spontaneously in non-aneurysmal vasculature [23], and, in the present cohort, by the persistence of the formation-associated geometric pattern even in aneurysms < 4 mm (Section 4.3). Under this framework, a wider opening angle and asymmetric branch calibers redirect the systolic inflow jet toward the bifurcation apex, elevating WSS and WSSG at the apical wall. Sustained abnormal WSS is sensed by endothelial mechanoreceptors and may trigger a maladaptive remodeling cascade—including endothelial dysfunction, matrix metalloproteinase upregulation, degradation of the internal elastic lamina, and phenotypic switching of medial smooth muscle cells toward a synthetic, pro-inflammatory state—that weakens the apical wall and favors aneurysm initiation [11,12,18,19,20,21]. A related hemodynamic–biological process may continue after initiation, as low and oscillatory WSS within the evolving sac has been associated with chronic inflammatory remodeling and aneurysm growth, offering a plausible mechanistic bridge between the formation-associated geometry identified here and the size-stratified findings in Section 4.3. We did not directly measure WSS or perform patient-specific computational hemodynamic simulations in this cohort, so this pathophysiological interpretation remains inferential and will require confirmation through direct hemodynamic modeling in future prospective studies.

ϕ1/D1 provides a compact size-normalized descriptor of the bifurcation opening relative to the parent caliber. It yielded the highest single-marker AUC, supporting its potential usefulness as a discriminator of aneurysm-bearing bifurcations [6,7]. However, when modeled with other correlated bifurcation parameters, its adjusted association was attenuated, likely reflecting collinearity with the opening angle and caliber-related variables. Although ϕ3 and the opening-to-caliber index showed *p* values close to the conventional threshold in the adjusted analyses, these findings should be interpreted as exploratory and do not establish independent associations after adjustment. In particular, the opening-to-caliber index may be considered a hypothesis-generating imaging marker that warrants external validation. A similar pattern of multicollinearity-related instability was observed for D1/D2, whose confidence interval was wide in the full model but narrowed substantially after stepwise variable removal. Although the direction of association was consistent across models, the magnitude and precision of the estimates were sensitive to model specifications, and this finding should be interpreted with this instability in mind. Beyond local hemodynamic and geometric factors, genetic susceptibility likely contributes to aneurysm formation, particularly among patients harboring multiple aneurysms. Familial intracranial aneurysm syndromes and systemic conditions associated with vessel wall weakness—such as autosomal dominant polycystic kidney disease and heritable connective tissue disorders—predispose to aneurysm formation at multiple, geometrically distinct sites, independent of local bifurcation configuration. In our cohort, a subset of patients harbored more than one MCA bifurcation aneurysm (see Limitations); although our GEE models statistically accounted for within-patient clustering, they cannot capture an underlying genetic predisposition that may act in parallel with, or interact with, the geometric factors identified here. Disentangling the relative contributions of local bifurcation geometry versus systemic genetic susceptibility will require future studies incorporating family history and genetic screening.

### 4.2. Rupture Subgroup: Sac-Level Morphology Predominates

In analyses restricted to aneurysm cases, rupture status was not significantly associated with bifurcation-level geometry, whereas sac-level morphology, particularly sac size, showed clearer separation between ruptured and unruptured aneurysms [1,2,4,13,14,15]. In our cohort, ruptured aneurysms were also associated with lower BMI and higher rates of current smoking; however, these patient-level differences should be interpreted cautiously given the modest rupture subgroup (n = 29) and mixed evidence regarding BMI and aneurysmal SAH risk [24,25]. Taken together, these findings suggest that rupture in this cohort was more closely related to sac-level morphology than to upstream bifurcation configuration. However, the absence of a detectable geometry–rupture association should not be interpreted as definitive, because the limited number of ruptured aneurysms may have reduced the ability to detect subtle geometry-related effects.

### 4.3. Size Subgroup: Geometry Patterns Across Aneurysm Size Strata

Across aneurysm size strata, larger aneurysms showed more pronounced aneurysm-presence-associated geometry, including wider bifurcation angles, more acute dominant-branch angles, and higher ϕ1/D1. These findings may reflect either pre-existing bifurcation geometry associated with a larger aneurysm phenotype or secondary remodeling as aneurysms enlarge; the present cross-sectional data cannot distinguish between these possibilities [8,9,23]. Notably, the association between a larger bifurcation opening angle and aneurysm presence remained detectable in the <4 mm subgroup, suggesting that the observed geometric differences are unlikely to be explained solely by aneurysm enlargement. Together, these findings support the hypothesis that bifurcation geometry may remain relevant not only to aneurysm presence but also to size-related aneurysm phenotype, although longitudinal studies are needed to determine whether these geometric features contribute to subsequent aneurysm growth. Prior studies have also suggested a dynamic relationship between parent artery geometry and aneurysm behavior: narrowing of the parent artery angle after coil embolization has been associated with aneurysm recurrence or growth, whereas straightening or widening of the parent artery angle after stent-assisted coil embolization may reduce recurrence risk [23]. Overall, these observations are consistent with the concept that vascular geometry can influence hemodynamic loading and aneurysm behavior [3,18,21,23].

### 4.4. Limitations

This study had some limitations. First, it was a single-center retrospective analysis of patients who underwent 3D rotational DSA at a tertiary center; therefore, referral and indication-related selection bias are possible, and generalizability to CTA- or MRA-based populations may be limited. Second, some patients had more than one MCA bifurcation aneurysm; GEE models accounted for patient-level clustering, but residual within-patient correlation cannot be excluded. Third, smoking was not included in the matching, and residual confounding by smoking or other unmeasured factors remains possible. Fourth, geometry-derived indices were used as indirect surrogates rather than direct patient-specific hemodynamic simulations. Fifth, the rupture subgroup was modest, and subgroup analyses were cross-sectional; therefore, subtle geometry rupture effects may have gone undetected, and causality for aneurysm enlargement cannot be inferred. Finally, although ϕ1/D1 showed the best single-marker discrimination, its generalizability and incremental value over established morphological parameters require prospective multicenter validation.

## 5. Conclusions

MCA bifurcation aneurysm presence was associated with a wider and more asymmetric bifurcation geometry, whereas rupture appeared to be more closely related to sac-level morphology than bifurcation geometry. Aneurysm-bearing bifurcations showed larger opening angles, more acute dominant-branch angles, lower parent-to-branch diameter ratios, and greater daughter-branch asymmetry than matched controls, and some of these geometric differences were more pronounced in larger aneurysms. These measurements may help characterize aneurysm-bearing bifurcations and, pending prospective validation, could complement size- and morphology-based assessments, particularly in small or borderline MCA bifurcation lesions.

## Data Availability

The datasets supporting the conclusions of this article are included within the article and its additional files.

## Supplementary Materials

The following supporting information can be downloaded at https://www.mdpi.com/article/10.3390/diagnostics16152312/s1. Supplementary Table S1. Definitions and measurement planes for bifurcation geometry variables; Supplementary Table S2. Definitions and measurement planes for sac-level morphology variables; Supplementary Table S3. Standardized mean differences after propensity-score matching; Supplementary Table S4. Operational definitions of vascular risk factors; Supplementary Table S5. Case-only baseline characteristics by rupture status; Supplementary Table S6. Case-only baseline clinical characteristics by aneurysm size; Supplementary Table S7. Stepwise GEE model for small aneurysms (<4 mm) versus controls; Supplementary Table S8. Sensitivity analyses for alternative aneurysm-formation models.

## Abbreviations

The following abbreviations are used in this manuscript:

ANOVA	Analysis of variance
aOR	Adjusted odds ratio
AUC	Area under the curve
BMI	Body mass index
CAD	Coronary artery disease
CFD	Computational fluid dynamics
CI	Confidence interval
CVD	Cerebrovascular disease
D1	Parent artery diameter (M1)
D2	Dominant daughter artery diameter (M2)
D3	Non-dominant daughter artery diameter (M2)
DSA	Digital subtraction angiography
HF	Heart failure
HTN	Hypertension
ICC	Intraclass correlation coefficient
IQR	Interquartile range
M1	First segment of the middle cerebral artery
M2	Second segment of the middle cerebral artery
MCA	Middle cerebral artery
ROC	Receiver operating characteristic
ROC-AUC	Area under the receiver operating characteristic curve
SAH	Subarachnoid hemorrhage
SMD	Standardized mean difference
ϕ1	Bifurcation opening angle
ϕ2	M1–dominant M2 branch angle
ϕ3	M1–non-dominant M2 branch angle
GEE	Generalized estimating equation
