# Supplementary material for: Middle Cerebral Artery Bifurcation Geometry and Aneurysm Formation: A Propensity Score-Matched 3D Rotational Morphometry Study
Source: Diagnostics (Basel). 2026 Jul 23;16(15):2312. doi: 10.3390/diagnostics16152312 (PMC13464841; doi:10.3390/diagnostics16152312)
Supplement: Supplementary file 1 [file diagnostics-16-02312-s001.zip › diagnostics-4404799-supplementary.pdf]

Supplementary Materials

Supplementary Table S1. Definitions and measurement planes for bifurcation geometry variables

Variable	Definition
Bifurcation angle (ϕ1, °)	The angle between the centerlines of the two M2 trunks at the bifurcation apex, measured in the plane containing both M2 centerlines.
Dominant branch angle (ϕ2, °)	The smaller lateral angle between the M1 trunk and the dominant M2 branch, measured in the plane containing the M1 and dominant M2 centerlines.
Non-dominant branch angle (ϕ3, °)	The larger lateral angle between the M1 trunk and the non-dominant M2 branch, measured in the plane containing the M1 and non-dominant M2 centerlines.
Parent diameter (D1, mm)	The luminal diameter of M1 was measured on a straight, non-tapering segment midway between the ICA bifurcation and the M1–M2 bifurcation, using a cross-section perpendicular to the M1 axis.
Dominant daughter diameter (D2, mm)	The luminal diameter of the dominant M2 branch measured 5 mm distal to the bifurcation apex on a cross-section perpendicular to the dominant M2 axis.
Non-dominant daughter diameter (D3, mm)	The luminal diameter of the non-dominant M2 branch measured 5 mm distal to the bifurcation apex on a cross-section perpendicular to the non-dominant M2 axis.
Parent-to-dominant diameter ratio (D1/D2)	The ratio of the parent M1 diameter to the dominant M2 diameter, derived from D1 and D2.
Parent-to–non-dominant diameter ratio (D1/D3)	The ratio of the parent M1 diameter to the non-dominant M2 diameter, derived from D1 and D3.
Lateral angle ratio (ϕ3/ϕ2)	The ratio of the non-dominant branch angle to the dominant branch angle, derived from ϕ3 and ϕ2.
Opening-per-caliber index (ϕ1/D1, °/mm)	The bifurcation opening angle normalized by the parent M1 diameter, derived from ϕ1 and D1.
Parent cross-sectional area (cm^2)	The M1 cross-sectional area calculated as $\pi \cdot (D1/2)^2$ using D1 measured on a cross-section perpendicular to the M1 axis.

Supplementary Table S2. Definitions and measurement planes for sac-level morphology variables

Variable	Definition
Maximum size (mm)	The maximum aneurysm diameter, defined as the largest dome-to-dome distance measured on the 3D sac surface.
Height maximum (mm)	The maximum sac height, measured as the distance from the neck plane to the dome tip along the aneurysm main axis.
Width maximum (mm)	The maximum dome width, measured perpendicular to the direction of the height (aneurysm main axis).
Neck diameter (mm)	The maximum neck width at the parent–sac interface, measured on the neck plane orthogonal to the parent vessel axis.
Aneurysm volume (mm^3)	The aneurysm sac volume, calculated from segmentation using a voxel-based method.
Convex-hull volume (mm^3)	The volume of the convex hull enclosing the aneurysm sac, computed from the convex-hull surface.
Convex-hull area (mm^2)	The surface area of the convex hull enclosing the aneurysm sac, computed from the convex-hull surface.
Flow angle (°)	The angle between the parent vessel axis and the aneurysm main axis, measured in the plane containing both axes.
Inclination angle (°)	The angle between the aneurysm main axis and the line perpendicular to the parent vessel axis, measured in the plane containing both axes.
Vessel angle (°)	The angle between the proximal and distal parent vessel centerline segments adjacent to the aneurysm.
Bleb formation	The presence of a discrete secondary outpouching (bleb) on the aneurysm surface, assessed on 3D DSA reconstruction.

Supplementary Table S3. Standardized mean differences after propensity-score matching

**Variable**	**SMD**	**Absolute SMD**
Age	0.006984	0.006984
BMI	0.059133	0.059133
HTN	0.022711	0.022711
Sex	−0.05512	0.055119

SMD, standardized mean difference

Supplementary Table S4. Operational definitions of vascular risk factors

Variable	SMD
Hypertension	Systolic blood pressure ≥140 mmHg or diastolic blood pressure ≥90 mmHg, or current use of antihypertensive medication
Diabetes mellitus	Fasting plasma glucose ≥126 mg/dL, HbA1c ≥6.5%, or current use of antidiabetic medication
Hyperlipidemia	Total cholesterol ≥240 mg/dL or current use of lipid-lowering therapy
Heart disease	Any documented arrhythmia (atrial fibrillation or atrial flutter), coronary artery disease, or heart failure
Family history of CVD	First-degree relative with myocardial infarction, ischemic stroke, or sudden cardiac death (men <55 years, women <65 years)
Family history of aneurysm	First-degree relative with an intracranial aneurysm or aneurysmal subarachnoid hemorrhage
Smoking status	Current: active cigarette smoking at index date
Drinking status	Current: any alcohol use in the past 12 months
Antiplatelet medication	Current use of aspirin, clopidogrel, prasugrel, or ticagrelor
Anticoagulant medication	Current use of warfarin or a direct oral anticoagulant
Lipid-lowering medication	Current use of statins, ezetimibe, or PCSK9 inhibitors

Supplementary Table S5. Case-only baseline characteristics by rupture status

Variable	Unruptured (n=104)	Ruptured (n=29)	p
Age (years)	60.38 ± 8.15	59.55 ± 10.71	0.700
Sex (Male)	32 (30.8)	13 (44.8)	0.233
BMI (kg/m²)	24.43 ± 3.71	21.99 ± 3.50	0.002
Hypertension (Yes)	62 (59.6)	13 (44.8)	0.227
Diabetes mellitus (Yes)	9 (8.7)	1 (3.4)	0.690
Hyperlipidemia (Yes)	22 (21.2)	4 (13.8)	0.536
Heart disease (Any)	6 (5.8)	2 (6.9)	0.764
Family Hx of CVD (Yes)	1 (1.0)	1 (3.4)	0.390
Family Hx of aneurysm (Yes)	2 (1.9)	1 (3.4)	0.525
Drinking – Current	36 (34.6)	16 (55.2)	0.073
Smoking – Current	25 (24.0)	14 (48.3)	0.021
Antiplatelet (Yes)	20 (19.2)	2 (6.9)	0.159
Anticoagulant	0 (0.0)	0 (0.0)	1.000
Lipid-lowering agent	22 (21.2)	4 (13.8)	0.536

Supplementary Table S6. Case-only baseline clinical characteristics by aneurysm size

Variable	Small (<4 mm) (n=35)	Medium (4–6.9 mm) (n=62)	Large (≥7 mm) (n=36)	p
Age	59.94 ± 8.62	59.55 ± 8.35	61.58 ± 9.53	0.567
Sex (%)				0.917
Male	11 (31.4)	21 (33.9)	13 (36.1)	
Female	24 (68.6)	41 (66.1)	23 (63.9)	
BMI	23.25 ± 2.81	24.03 ± 4.02	24.31 ± 4.20	0.367
HTN (%)	17 (48.6)	34 (54.8)	24 (66.7)	0.290
Diabetes mellitus (%)	2 (5.7)	5 (8.1)	3 (8.3)	1.000
Hyperlipidemia (%)	3 (8.6)	17 (27.4)	6 (16.7)	0.070
Heart disease (%)				0.330
Arrhythmia	1 (2.9)	0 (0.0)	0 (0.0)	
CAD	2 (5.7)	1 (1.6)	3 (8.3)	
HF	0 (0.0)	1 (1.6)	0 (0.0)	
Family Hx of CVD (%)	0 (0.0)	1 (1.6)	1 (2.8)	1.000
Family Hx of aneurysm (%)	0 (0.0)	3 (4.8)	0 (0.0)	0.344
Drinking (%)	14 (40.0)	25 (40.3)	13 (36.1)	0.930
Smoking (%)	11 (31.4)	16 (25.8)	12 (33.3)	0.696
Antiplatelet (%)	5 (14.3)	9 (14.5)	8 (22.2)	0.561
Anticoagulant (%)	0 (0.0)	0 (0.0)	0 (0.0)	1.000
Lipid-lowering agent (%)	3 (8.6)	16 (25.8)	7 (19.4)	0.121

Supplementary Table S7. Stepwise GEE model for small aneurysms (<4 mm) versus controls

**Predictor (fitted unit)**	**Final stepwise GEE aOR (95% CI)**	**P**
Bifurcation angle, ϕ1 (per 1°)	1.10 (1.04–1.17)	0.002
M1–dominant M2 angle, ϕ2 (per 1°)	Not retained in the final model	—
M1–non-dominant M2 angle, ϕ3 (per 1°)	Not retained in the final model	—
D1/D2 (per 1.0)	Not retained in the final model	—

Supplementary Table S8. Sensitivity analyses for alternative aneurysm-formation models

**Model**	**Variable**	**OR (95% Cl)**	**P**
Smoking-adjusted	ϕ1	1.07 (1.05–1.10)	< 0.001
Smoking-adjusted	ϕ2	0.98 (0.97–0.99)	0.003
Smoking-adjusted	ϕ3	0.99 (0.97–1.00)	0.041
Smoking-adjusted	Cross-sectional area	0.00 (0.00–0.00)	< 0.001
Smoking-adjusted	D1/D2	0.14 (0.02–0.80)	0.027
Smoking-adjusted	ϕ1/D1	0.95 (0.92–0.99)	0.025
Smoking-adjusted	Current smoking	1.09 (0.52–2.27)	0.817
One per patient (GLM)	ϕ1	1.07 (1.04–1.10)	< 0.001
One per patient (GLM)	ϕ2	0.98 (0.96–1.00)	0.010
One per patient (GLM)	ϕ3	0.98 (0.97–1.00)	0.035
One per patient (GLM)	Cross-sectional area	0.00 (0.00–0.00)	0.001
One per patient (GLM)	D1/D2	0.14 (0.03–0.76)	0.023
One per patient (GLM)	ϕ1/D1	0.95 (0.91–0.99)	0.016
ϕ1/D1 alternative	ϕ1/D1	1.08 (1.05–1.11)	< 0.001
ϕ1/D1 alternative	ϕ2	0.98 (0.97–0.99)	0.002
ϕ1/D1 alternative	ϕ3	0.99 (0.98–1.00)	0.045
ϕ1/D1 alternative	Cross-sectional area	0.00 (0.00–0.00)	< 0.001
ϕ1/D1 alternative	D1/D2	0.15 (0.03–0.80)	0.027
